# Structural and immunological differences in *Plasmodium falciparum* sexual stage transmission-blocking vaccines comprised of Pfs25-EPA nanoparticles

**DOI:** 10.1038/s41541-023-00655-5

**Published:** 2023-04-15

**Authors:** Nicholas J. MacDonald, Kavita Singh, Karine Reiter, Vu Nguyen, Richard Shimp, Apostolos G. Gittis, Beth Chen, Martin Burkhardt, Baoshan Zhang, Zhixiong Wang, Raul Herrera, Mackenzie Moler, Duck-Yeon Lee, Sachy Orr-Gonzalez, Jessica Herrod, Lynn E. Lambert, Kelly M. Rausch, Olga Muratova, David S. Jones, Yimin Wu, Albert J. Jin, David N. Garboczi, Patrick E. Duffy, David L. Narum

**Affiliations:** 1 Laboratory of Malaria Immunology and Vaccinology, National Institute of Allergy and Infectious Diseases, National Institutes of Health, 29 Lincoln Drive, Bethesda, MD 20892 USA; 2 Structural Biology Section, Research Technologies Branch, National Institute of Allergy and Infectious Diseases, National Institutes of Health, 29 Lincoln Drive, Bethesda, MD 20892 USA; 3 Vaccine Research Center, National Institute of Allergy and Infectious Diseases, National Institutes of Health, Bethesda, MD 20814 USA; 4 Laboratory of Cellular Imaging and Macromolecular Biophysics, National Institute of Biomedical Imaging and Bioengineering, National Institutes of Health, Bethesda, Maryland 20892 USA; 5 National Heart, Lung, and Blood Institute, Bethesda, MD 20814 USA

**Keywords:** Protein vaccines, Malaria

## Abstract

Development of a malaria vaccine that blocks transmission of different parasite stages to humans and mosquitoes is considered critical for elimination efforts. A vaccine using Pfs25, a protein on the surface of zygotes and ookinetes, is under investigation as a transmission-blocking vaccine (TBV) that would interrupt parasite passage from mosquitoes to humans. The most extensively studied Pfs25 TBVs use *Pichia pastoris*-produced recombinant forms of Pfs25, chemically conjugated to a recombinant carrier protein, ExoProtein A (EPA). The recombinant form of Pfs25 first used in humans was identified as Pfs25H, which contained a total of 14 heterologous amino acid residues located at the amino- and carboxyl-termini including a His6 affinity tag. A second recombinant Pfs25, identified as Pfs25M, was produced to remove the heterologous amino acid residues and conjugated to EPA (Pfs25M-EPA). Here, monomeric Pfs25M was characterized biochemically and biophysically for identity, purity, and integrity including protein structure to assess its comparability with Pfs25H. Although the biological activities of Pfs25H and Pfs25M, whether generated by monomeric forms or conjugated nanoparticles, appeared similar, fine-mapping studies with two transmission-blocking monoclonal antibodies detected structural and immunological differences. In addition, evaluation of antisera generated against conjugated Pfs25H or Pfs25M nanoparticles in nonhuman primates identified polyclonal IgG that recognized these structural differences.

## Introduction

Malaria continues to be a disease of global concern. Unfortunately, recent gains in reducing the incidence of malaria appear at risk since global mortality has risen for the past several years^1^. Two biological bottlenecks in the parasite life cycle, each having a reduced number of parasites compared to the stage responsible for clinical disease, have been considered as more robust targets in *falciparum* malaria, the parasite species responsible for the greatest morbidity and mortality. The first bottleneck is the *Plasmodium* pre-erythrocytic stage; the world’s first malaria vaccine RTS,S (Mosquirix) acts against this stage by targeting the abundant circumsporozoite protein (CSP) on the sporozoite surface, and was recently recommended for use by WHO^2^. The second bottleneck occurs during *Plasmodium* sexual stage development in the mosquito; a vaccine disrupting sexual development aims to block subsequent transmission to the human host. Although RTS,S is effective, vaccine efficacy is less than 40%, leaving opportunities for improvement. Vaccine efficacy may be improved by using alternative boundaries of the CSP, as RTS,S comprises only the carboxyl half of CSP, or by combining CSP with additional vaccine antigens. Our efforts have focused on developing a transmission-blocking vaccine (TBV), including candidates that target a 25 kDa *P. falciparum* sexual stage protein identified as Pfs25, generated in the methylotrophic *Pichia pastoris* expression platform^3,4^. Orthologues of Pfs25 have been identified and in particular, the *P. vivax* sexual stage protein Pvs25 is considered as a TBV candidate^5^. A combination vaccine that protects against both *P. falciparum* and *P. vivax* would be a significant contribution towards malaria control.

Pfs25 is displayed on the surface of zygotes and ookinetes, two parasite stages found within the mosquito midgut^6^. Several clinical trials have been conducted using two recombinant forms of Pfs25 produced in *P. pastoris*^7,8^. The initial recombinant form used in clinical trials is Pfs25H, which contains a total of 14 heterologous amino acid residues—six located at the amino terminus and eight at the carboxy-terminus^7,8^. To better prepare for late-phase clinical trials, a second recombinant Pfs25 protein, called Pfs25M, was produced containing no heterologous amino- or carboxyl-terminal amino acid residues^9^. Each of these Pfs25 recombinant forms was conjugated to the carrier protein, ExoProtein A, a detoxified form of the *Pseudomonas aeruginosa* Exotoxin A, to form a nanoparticle which enhances the immunogenicity of Pfs25^10,11^. In human vaccine trials, both Pfs25H-EPA and Pfs25M-EPA were safe and immunogenic, and induced transmission-blocking antibodies following four doses using an ex vivo mosquito feeding assay^7,9,12^.

Here we report full biochemical and biophysical characterization of Pfs25M and Pfs25M-EPA, including Pfs25M crystallization in complex with a Fab of the transmission-blocking antibody, 1G2. Furthermore, we demonstrate that different protein modifications impacted the 1) binding of two transmission-blocking monoclonal antibodies (mAbs) against the monomeric proteins, and 2) specificity of antibody responses (i.e., IgG) in rhesus monkeys following active immunization.

## Results

### Production and characterization of recombinant Pfs25M

**Fig. 1:**
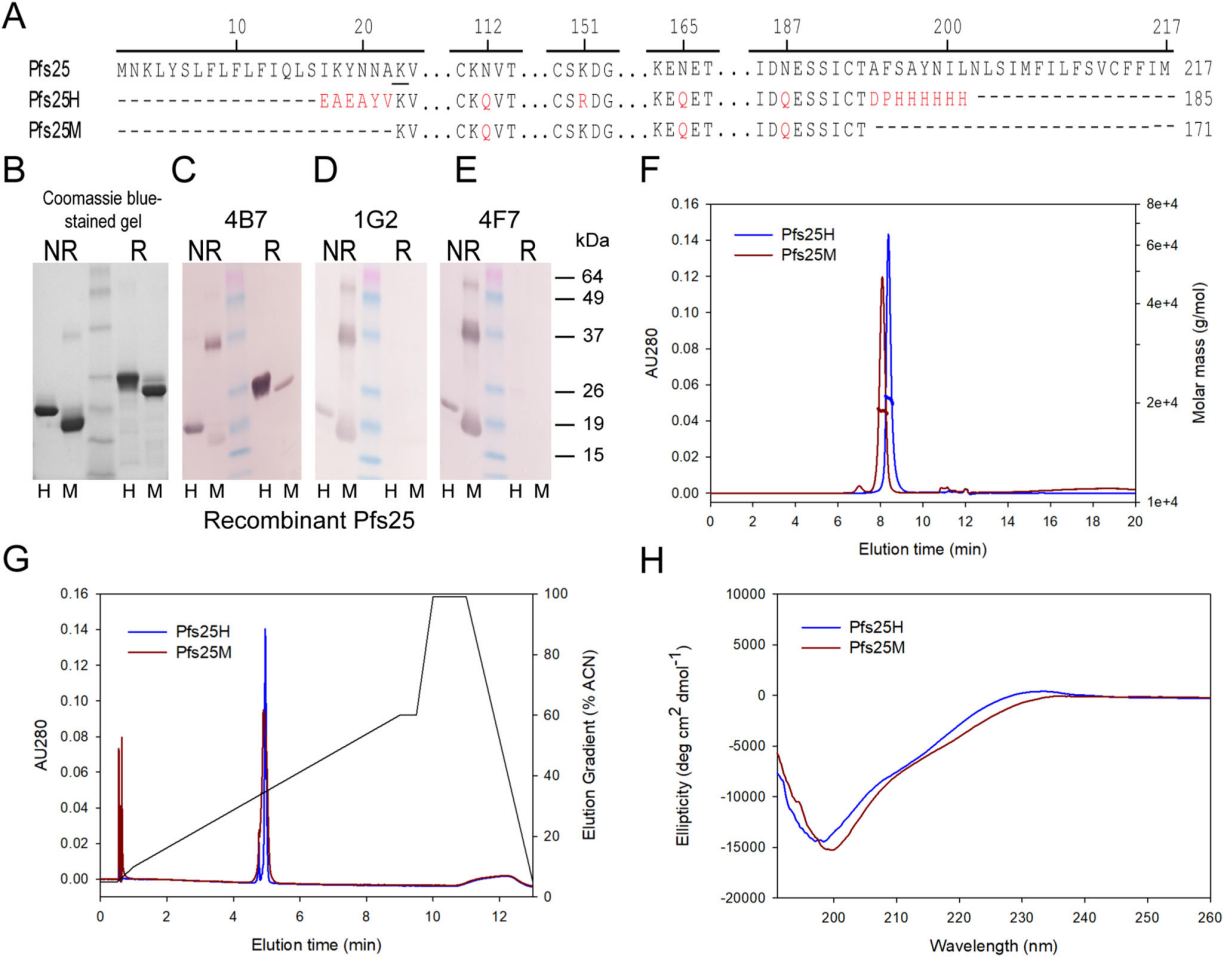
Production and characterization of Pfs25M and Pfs25H. Abbreviated native Pfs25, Pfs25H, and Pfs25M protein sequences. Differences (in red) between Pfs25H and Pfs25M are discussed in the text (**A**). Evaluation of purified recombinant Pfs25H (H) and Pfs25M (M) under non-reduced (NR) and reduced (R) conditions by Coomassie blue stained SDS-PAGE (**B**) and immunoblots (**C**–**E**). Biophysical characterizations of Pfs25M and Pfs25H by analytical SEC-MALS (**F**), reversed-phase UPLC (**G**) and far-UV CD spectroscopy (**H**). The panel of Pfs25-specific mAbs used were 4B7 (**C**), 1G2 (**D**) and 4F7 (**E**). Wild-type Pfs25 sequence accession number is P13829 (previously AAN35500.1). The amino acid (K23) following cleavage of the signal sequence is underlined.

Pfs25M contained several changes compared to the previously reported Pfs25H^4^. The *P. pastoris*-expressed Pfs25M was modified to exclude the heterologous N-terminal amino acid residues EAEAYV, which were originally included to improve cleavage of the N-terminal yeast α-factor signal sequence in Pfs25H (Fig. 1A, red). This change was confirmed in recombinant Pfs25M by Edman degradation protein sequencing which showed the expected N-terminal sequence KVTVDTVCKRGF. The C-terminal heterologous residues of Pfs25H were omitted, which removed a purification His6 tag and two adjacent residues (Fig. 1A, red). As was done in the design of Pfs25H, the three predicted N-linked glycosylation sites of Pfs25M were mutated from NxS/T to QxS/T to avoid N-linked glycosylation (Fig. 1A, red). A mutation, R151, observed in the Pfs25H synthetic gene was changed to the wild-type K151 in Pfs25M. Pfs25M starts at K23 and terminates at T193 based on the 3D7 Pfs25 sequence. Disulfide bond formation in the four EGF-like domains in Pfs25M and Pfs25H was confirmed by an observed mobility shift by Coomassie blue stained SDS-PAGE under non-reduced versus reduced conditions (Fig. 1B, raw images in Supplementary Fig. 1) and by intact mass spectrometry. The observed intact masses of 18,712 Da for Pfs25M and of 20,438 Da for Pfs25H were within 1 Da of the predicted masses of 18,713 Da and 20,438 Da, respectively. Shifts in the intact masses of ~22 Da were observed upon reduction of the recombinant proteins, as predicted for the presence of eleven disulfide bonds.

To assess protein conformation, a panel of transmission-blocking mAbs was used for blotting against recombinant Pfs25H and Pfs25M (Fig. 1C–E, raw images in Supplementary Fig. 1). Conformation-dependent mAbs 1G2 and 4F7 only recognized the non-reduced forms (monomer and some dimer) consistent with their requirement for disulfide bonds to be present^13^, while mAb 4B7, which in its initial reporting^14^ strongly recognized non-reduced native Pfs25 and weakly with reduced Pfs25, recognized Pfs25H under non-reduced conditions as observed previously^4^. The reaction of mAb 4B7 with Pfs25M under non-reduced conditions was weaker than expected when compared to its reactivity with Pfs25H (Fig. 1C). MAbs 1G2 and 4F7 did not react with Pfs25H or Pfs25M (Fig. 1D–E). Analyses by analytical size exclusion chromatography with detection by in-line multi-angle light scattering (SEC-MALS) demonstrated that Pfs25M and Pfs25H were principally monomeric in solution (>95%) with an observed molar mass of 17.9 kDa and 19.8 kDa, respectively (Fig. 1F). By reversed-phase (RP)-UPLC Pfs25M and Pfs25H were resolved as single peaks with similar retention times (4.4867 and 4.490 min, respectively) (Fig. 1G). Far-UV circular dichroism (CD) spectra demonstrated that Pfs25M has a similar secondary structure to Pfs25H (Fig. 1H). We also evaluated the thermal stability of Pfs25M and Pfs25H using a temperature ramp and measured the change in secondary structure by far-UV CD spectroscopy. A change in the ellipticity was observed between 55 °C and 60 °C for both forms. After complete denaturation at 80 °C and cooling to 20 °C, CD spectra nearly identical to the before-heating spectra were obtained, indicating that their secondary structures refolded (Supplementary Fig. 2). Such reversibility after denaturation demonstrates high thermal stability, which is desirable for a vaccine component. The endotoxin levels were all below 10 endotoxin units per mg protein and the concentration of host cell proteins, using a process-specific immunoblot assay, was less than the limit of detection (<0.001% w/w).

### Immunogenicity of recombinant Pfs25M and Pfs25H in rabbits, and transmission-reducing activity (TRA) of antisera

Pfs25M and Pfs25H, formulated in a water-in-oil adjuvant, were each used as an immunogen (or antigen for immunization) in two rabbits. The ELISA titers for Pfs25M as an immunogen were 236,217 and 90,658 using Pfs25M as plate antigen and 190,952 and 88,905 using Pfs25H as plate antigen. The ELISA titers for Pfs25H as an immunogen were 39,019 and 91,165 and 25,178 and 91,571 against Pfs25H or Pfs25M as plate antigens, respectively. Antibodies generated against either Pfs25 molecule reacted well against both Pfs25 molecules.

**Fig. 2:**
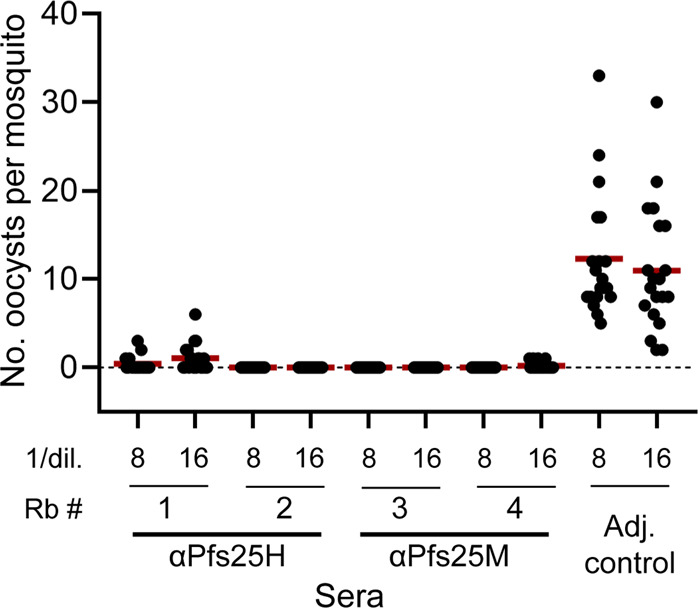
Evaluation of the transmission-blocking activity of rabbit antisera raised against Pfs25H or Pfs25M compared to an adjuvant control using the standard membrane feeding assay (SMFA). Shown are the number of oocysts in a minimum of 20 mosquitoes per test sample (•) and the average number of oocysts (**—**). The average inhibition of oocyst density (TRA) in percent was: Rb1 97.1, 90.7; Rb2 100, 100; Rb3 100, 100; and Rb4 100, 98.2 against the appropriate adjuvant controls (0%). All sera were heat-inactivated and dilutions (1/8 and 1/16) prepared that included supplement with human serum with active complement. The *P. falciparum* NF54 strain was used in the ex vivo SMFA.

The rabbit antisera generated against each of the two recombinant Pfs25 proteins (3D7 strain) contained transmission-reducing activity (TRA) assessed as the reduction in the mean oocyst density compared to an adjuvant only control serum, reported as a percent in the standard membrane feeding assay (SMFA)^13^. A comparison at 1:8 and 1:16 dilutions showed no marked differences in TRA (Fig. 2). Complete transmission-blocking activity was observed by all rabbit sera at a 1:4 dilution in the SMFA (Supplementary Fig. 3).

### Pfs25M and Pfs25H binding in solution by capture ELISA and isothermal titration calorimetry (ITC)

**Fig. 3:**
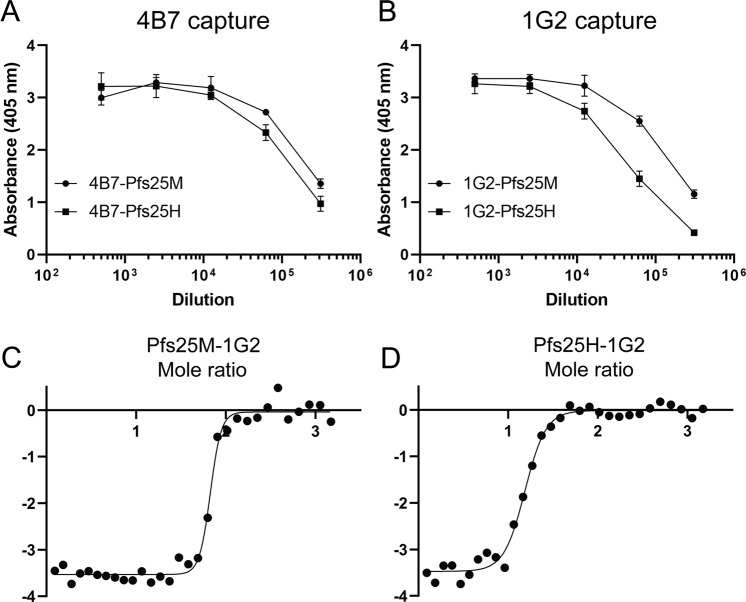
Evaluation of Pfs25 specific mAbs 4B7 or 1G2 binding Pfs25H and Pfs25M in solution by solid phase or by isothermal titration calorimetry with 1G2. MAbs 4B7 **(A**) and 1G2 (**B**) were plated and incubated with Pfs25H or Pfs25M in solution followed by detection with rabbit Pfs25M specific sera and a species-specific secondary reagent. Isothermal titration calorimetry for mAb 1G2 binding to Pfs25M (**C**) or Pfs25H (**D**). The binding constants, Kd, for Pfs25M and Pfs25H were 6.3 × 10^−9^ and 4.1 × 10^−8^, respectively. Symbols and error bars in A and B represent the mean and standard deviation.

To further understand the absence of reactivity to Pfs25M by mAb 4B7 on immunoblots under non-reduced conditions (Fig. 1C), a mAb capture ELISA was developed to evaluate antibody binding of Pfs25M and Pfs25H in solution. The conformation-dependent mAb 1G2 was included as a positive control. Prior to assessing the binding, we confirmed that both mAbs had similar TRA (Supplementary Fig. 4). In solution, mAb 4B7 bound Pfs25M and Pfs25H similarly (Fig. 3A). In the ELISA, however, mAb 1G2 showed nearly a 5-fold difference in binding Pfs25M compared to Pfs25H, within the linear range of the titration curves at an absorbance of approximately 2 (Fig. 3B). To understand these differences, ITC was used to estimate the binding constants for 1G2 in solution in combination with Pfs25M or Pfs25H. The results, which were in agreement with those observed by ELISA, showed a difference in their binding kinetics by a factor of 6.5. The lower Kd for binding 1G2 by Pfs25M, 6.3 × 10^−9 ^M, was indicative of a higher affinity binding when compared to the Kd for binding 1G2 by Pfs25H of 4.1 × 10^−8 ^M (Fig. 3C, D).

### Location of the 1G2 epitope on Pfs25

**Fig. 4:**
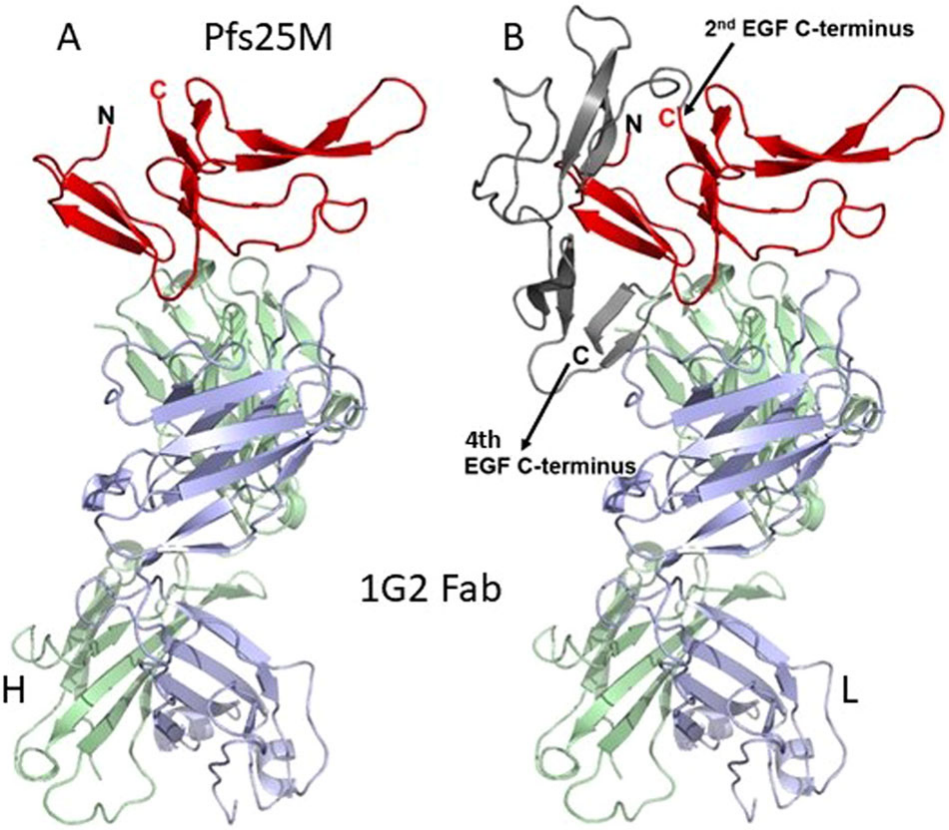
Structure of Pfs25M in complex with the 1G2 Fab. In (**A**), is the structure of the first two epidermal growth factors (EGF) like domains of Pfs25M (red). The 1G2 Fab binds to the two EGF-like domains with its heavy chain (H, green) making contacts with residues of EGF-like domain 1 and its light chain (L, blue) making contacts with EGF-like domain 2. In (**B**), similar to (**A**), but with domains 3 and 4 (gray) from the Pfs25 structure from PDB code: 6PHB^15^ overlaid on the first two EGF-like domains (red) from the 1G2 structure. EGF-like domains 3 and 4 (gray) are nestled with domains 1 and 2 to form a roughly triangular shape. The C-termini of EGF domains 2 and 4 are labeled.

The 1G2 epitope was found by co-crystallizing Pfs25M with the 1G2 Fab and determining the structure of the complex (Fig. 4A). The crystal structure revealed that the 1G2 Fab binds to its epitope located in the first and second EGF-like domains of Pfs25M. It also showed that Pfs25M had been cleaved between the second and third domain after residue N108, by an unidentified protease that was possibly introduced during the preparation of the complex or the crystallization experiment. The 1G2 IgG had been treated with the highly specific tobacco etch virus protease to produce the Fabs from the IgG, and the protease was removed before forming the complex of Fab with Pfs25M. When the database Pfs25 structure 6PHB chain D (Fig. 4B) was overlaid onto the first two domains of Pfs25M present in the 1G2 complex structure, the root mean square deviation (rmsd) was 0.9 angstroms between 79 alpha-carbon pairs. Such a low rmsd indicates that domains 1 and 2 of the 1G2/Pfs25M structure are virtually identical to those in the four-domain database Pfs25 structure 6PHB^15^.

### Production, characterization, and immunogenicity of Pfs25M-EPA conjugated nanoparticles

Pfs25M was chemically conjugated with the carrier protein ExoProtein A (EPA) using a thioether crosslinker as reported for Pfs25H^11^. The Pfs25M-EPA conjugated nanoparticle was eluted as essentially a single peak by reversed-phase-UPLC and was >95% a single large peak by analytical size exclusion chromatography (Supplementary Fig. 5A–C). The average molar mass was 373 kDa and the hydrodynamic radius ranged from 7–18 nm with a weight-average nanoparticle radius of 9.8 nm. The particle nature of the conjugate was further confirmed by atomic force microscopy (AFM) under dehydration (Supplementary Fig. 4D, E) and in buffer solution (Supplementary Fig. 6). The particle size distribution ranged from 8 to 19 nm under dehydration conditions and was consistent with the particle sizes observed in solution (Supplementary Fig. 5C and Supplementary Fig. 6A–D). Complex internal constructs were revealed under dehydration and high AFM resolution (Supplementary Fig. 5D, E). In solution, Pfs25M-EPA appeared as more spherical but soft nanoparticles with size-dependent mechanical compliance around the value of 5 nm/nN (Supplementary Fig. 6E, F).

To compare the immunogenicity of conjugated Pfs25M-EPA with that of Pfs25H-EPA, a mouse immunogenicity study was performed to compare antibody responses induced using different doses, ranging from 0.003 µg to 0.3 µg, of Pfs25M-EPA or Pfs25H-EPA administered on days 0 and 28. Both conjugates were formulated on Alhydrogel® and antisera were evaluated on day 42. An example of comparative immunogenicity demonstrated no significant differences in the ELISA units using a two-way ANOVA (*p* = 0.31) (Supplementary Fig. 7).

**Fig. 5:**
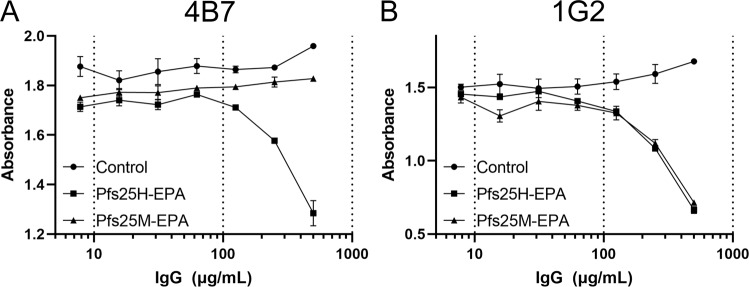
Competition of pooled immune rhesus IgG with Pfs25 specific mAbs 4B7 and 1G2. Competition ELISAs with HRP-labeled 4B7 (**A**) and 1G2 (**B**), using Pfs25M as the plated antigen. The concentrations of 4B7 and 1G2 were kept constant, while the pooled rhesus IgG concentration was varied to examine competition between IgG and mAbs. IgGs (“Control”, “Pfs25H-EPA”, “Pfs25M-EPA”) were purified from pooled pre-immune sera or from pooled antisera collected two weeks following a third dose. Rhesus were immunized with Pfs25H-EPA or Pfs25M-EPA formulated with Alhydrogel®. Symbols and error bars represent the mean and standard deviation.

Formulations of Pfs25M-EPA/Alhydrogel® and Pfs25H-EPA/Alhydrogel® were used to immunize rhesus monkeys using the standard human dosage of 47 µg Pfs25M or Pfs25H on days 0, 56, and 112 (Pfs25H-EPA) or 168 (Pfs25M-EPA). Similar geometric mean ELISA units of 14,234 for Pfs25M-EPA and 9697 for Pfs25H-EPA were obtained following the third immunization. TRA levels by the SMFA of 39.9% for Pfs25M and 38.4% for Pfs25H were also similar against NF54 parasites (Supplementary Fig. 8). Given the comparability of these conjugates and IgG responses, IgG was purified from pooled rhesus sera to perform competition ELISAs against enzymatically labeled 4B7 or 1G2. The results showed that immunization with Pfs25H-EPA produced polyclonal IgG that competed against both 4B7 and 1G2 (Fig. 5B), while immunization with Pfs25M-EPA produced polyclonal IgG that did not compete with 4B7, but only with 1G2 (Fig. 5A).

### Comparison of the 1G2 epitope with other functional mAbs, and enhanced TRA by mAb combination

**Fig. 6:**
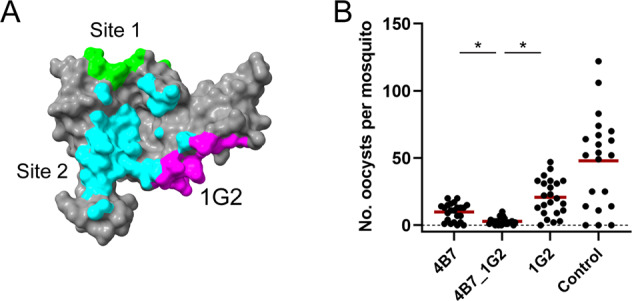
Mapping of 1G2 and other reported transmission-blocking mAbs and enhanced TRA with mAb combination. The mAb 1G2 epitope (magenta) is shown in context to other transmission-blocking regions identified as Site 1 (green) and Site 2 (cyan)^14,15^, respectively. (**A**). Site 1 overlaps with the binding site of mAb 4B7^14^. The combination of mAbs 1G2 and 4B7 shows enhanced TRA compared to each alone (**B**). The average TRA (reduction of oocyst density) was 81.6% (4B7 alone), 60.6% (1G2 alone), and 94.6% (combination of 4B7 and 1G2) at 158 µg/mL, 149 µg/mL, or 158/149 µg/mL, respectively, compared to the control (mAb 48F8 at 321 µg/mL). A comparison of the TRA between 4B7 or 1G2 with 4B7 and 1G2 in combination, showed significant differences (*, *p* < 0.001) using a zero-inflated negative binomial model with an interaction term. P-values are from post-hoc linear contrasts.

The 1G2 epitope was mapped in relationship to other reported murine and human anti-Pfs25 mAbs that show TRA (Fig. 6A). The ten antibody epitopes on Pfs25 that have been identified by crystal structures cluster into mainly two sites on Pfs25^14,15^. Several other antibodies have been mapped to those sites by competitive binding assays^15^. The 1G2 epitope is located in a third site on EGF-like domains 1 and 2, distal to the other two regions identified as site 1 and site 2 (Fig. 6A). The 1G2 epitope partially overlaps the reported epitope of the mAb 2530, which is also on domains 1 and 2^15^.

We evaluated whether 1G2 would show enhanced biological activity when combined with another mAb recognizing a different region of Pfs25. We selected to use the site 1 specific mAb 4B7 alone and in combination with 1G2 at concentrations of about 0.1 mg/mL that would allow for TRA without complete blocking (Supplementary Fig. 4). We observed that the combination of 1G2 and 4B7 provided greater TRA (mean 94.6%) compared to the TRA of either mAb alone (1G2, 60.6% and 4B7, 81.6%, *p* < 0.001) (Fig. 6B).

## Discussion

The *P. falciparum* sexual stage protein Pfs25 has been a leading malaria transmission-blocking vaccine (TBV) candidate for several decades. Disrupting transmission to the mosquito host as well as the human host holds promise for targeting the two bottlenecks in the development cycle of malaria. We have conducted several clinical trials with recombinant forms of Pfs25, identified as Pfs25H^12^ or Pfs25M^9^. Pfs25H has been evaluated using Montanide ISA51 as a soluble monomer^8^ and chemically conjugated with a non-toxic carrier protein, ExoProtein A (EPA) with Alhydrogel®^7^. In a preclinical study in mice, Radtke et al.^16^ demonstrated significant improvement in humoral and cellular responses following conjugation with EPA as a carrier protein, that included the generation of T follicular helper cells.

Pfs25H contains heterologous amino acid residues at both the N- and C-termini including a C-terminal 6-histidine affinity tag. Since there is a reasonable concern regarding the use of a 6-histidine fusion tag in a commercial vaccine and in late-stage clinical trials, and that it was not essential, we produced recombinant Pfs25M which contains no heterologous amino acid residues at the N- or C-termini. Three putative N-linked glycosylation sites were removed by mutation as with Pfs25H. Here we examined Pfs25H and Pfs25M to compare their biophysical, antigenic, and, when conjugated to a carrier protein, immunogenic characteristics.

Full-biochemical and biophysical characterizations demonstrated that Pfs25M was analytically highly comparable to Pfs25H. There was a slight difference in the retention time on the reversed-phase-UPLC column which is likely due to 7.5% reduction in the length of the Pfs25M sequence. Their solubilities as monomeric proteins appeared similar by analytical sizing and in general, their ellipticity profiles were similar. Paired rabbit immunizations produced similar antibody titers, that demonstrated good TRA for each immunogen. One unique difference within the monomeric forms was observed by Western blot in which mAb 4B7, a well-established Pfs25 specific functional mAb failed to bind to Pfs25M under non-reduced conditions while it bound to Pfs25H. Mab 4B7 reacted with both Pfs25M and Pfs25H under reduced conditions, which is consistent with our previous observation^4^ that 4B7 recognizes a cryptic linear epitope^3^. Two other mAbs (1G2 and 4F7) that are active in TRA, recognized conformation-dependent epitopes, as expected^13^.

The murine mAb 4B7 was developed by a combination of immunizing with a recombinant *Saccharomyces cerevisiae* Pfs25 with modified N- and C-termini and exposure to parasite extracts containing native Pfs25^17^. We were interested to better understand the absence of 4B7 binding to Pfs25M by Western blot under non-reduced conditions. We assessed the binding of Pfs25M and Pfs25H in solution, using a capture ELISA. MAb 1G2 was included in the study design as a positive control. In solution, 4B7 bound Pfs25M and Pfs25H in a similar manner; only a minor difference in the OD was observed within the linear range of their respective curves. In contrast, a five- to seven-fold difference was observed between 1G2 binding Pfs25M compared to Pfs25H. To assess whether these binding differences were significant, we established the binding constants by ITC for 1G2 with Pfs25M and Pfs25H. The binding constants (Kd) of 6.3 × 10^−9^ for Pfs25M and of 4.1 × 10^−8^ for Pfs25H, differ by ~6.5-fold. These results strongly suggest the position of the modified or unmodified N- and C-termini of Pfs25H or Pfs25M (see Supplementary Fig. 9) impact the epitope display such that 4B7 solely recognizes Pfs25H in non-reduced Western blot while 1G2 binds Pfs25M with greater avidity, even though rabbit antisera to the two antigens display similar TRA.

We co-crystallized a recombinant 1G2-Fab with Pfs25M and identified the location of the 1G2 epitope on domains 1 and 2 of Pfs25M. We were surprised that Pfs25M in the crystal had lost its domains 3 and 4. An unknown protease (or proteases) was presumably introduced in the formation of the complex with the Fab or in the crystallization experiments. As a vaccine candidate, Pfs25M itself had been subjected to rigorous tests of its stability and level of impurities, which indicated a high stability and an undetectable level of impurities. The new C-terminus at N108 is located immediately after domain 2, in the 4-residue loop CIPNEC between C105 of domain 2 and C110 of domain 3. In our crystal, domains 1 and 2 were stable to proteolytic degradation, but domains 3 and 4 were not.

Several natural sequence variants of Pfs25 have been reported^18,19^ and we wondered if Pfs25 sequence variants might affect 1G2 binding. Of the twelve sites reported, four sites, R32K, L42M, L63V, and K64R, are in domains 1 and 2 of Pfs25M and Pfs25H, where 1G2 binds. R32 is located at the edge of the 1G2 epitope, and it appears that the conservative change R32K would be tolerated. L42 does not contact 1G2. L63 makes slight contact with 1G2 and the conservative change L63V is unlikely to affect 1G2 binding. K64 also makes slight contact with the edge of the 1G2 binding area and the conservative change K64R likely would not influence 1G2 binding.

Using the Pfs25H-EPA conjugation platform^11^, a Pfs25M-EPA conjugate was produced and shown to have similar biochemical and biophysical properties, including similar immunogenicity in mice, and similar TRA in rhesus monkeys although the activity level was modest as has been previously reported^9^. In phase 1 human trials, Pfs25H-EPA and Pfs25M-EPA formulated on Alhydrogel® performed similarly in independent US studies^7,9^. Additional human phase 1 studies are ongoing with Pfs25M-EPA formulated with AS01 which contains two immunostimulants MPL and QS-21 (Clinicaltrials.gov Identifier: NCT02942277). Despite their similarity in human trials with Alhydrogel®, we used pooled rhesus monkey antisera to purify IgG to investigate whether conjugated Pfs25M or Pfs25H impacted the specificity of the IgG antibody response with respect to the 4B7 and 1G2 epitopes. A change in the 4B7 binding curve was noted with Pfs25H-EPA specific IgG indicating a difference in the specificity of the antibody response against domain 3 which packs in proximity to the amino-terminus within domain 1 (Supplementary Fig. 9). Interestingly, no differences were observed for competition with 1G2 binding.

A strategy of quality-by-design to develop well-characterized investigational vaccines provides a strong basis for broader assessments of preclinical and clinical results. The leading position of Pfs25 as a TBV candidate was only recently challenged following the scaled development of another TBV candidate, Pfs230D1M^20^. Pfs230D1M is comprised of domain 1 from the *P. falciparum* 230 kDa sexual stage protein^20^ also expressed in *P. pastoris* without any heterologous amino acid residues. Given the breadth of the biochemical, biophysical, and immunological characterizations, it is possible to compare their strengths and weaknesses for use as a TBV. Recombinant Pfs230D1M had the biophysical characteristics suitable for using our chemical conjugation platform with EPA, and conjugated Pfs230D1-EPA generated a superior transmission-blocking activity compared to Pfs25M-EPA when formulated on Alhydrogel®^9^. Furthermore, no increase in TRA was observed by active immunization with both Pfs230D1 and Pfs25M conjugates^9^ or by mixing combinations of Pfs25 and Pfs230D1 specific mAbs^21^. Another protein found on the surface of gametes, Pfs48/45 appears to be a better candidate for a two-component TBV, if necessary, since a combination of two functional mAbs against Pfs230D1 and Pfs48/45 demonstrated improved TRA versus either alone^21^.

In summary, we produced and characterized Pfs25M which is a highly comparable monomeric form to that of Pfs25H. We demonstrate that it behaves similarly both as a monomer and as a conjugated nanoparticle including its immunological characteristics in mice, monkeys and in limited human trials. Whether the discrete differences in specificity of antibody responses (IgG) noted here in rhesus monkeys are similar in human vaccinees has not been investigated. Although we observed minor differences between these two comparable recombinant proteins, our work strongly supports the design of a candidate that is suitable for both early and late-phase clinical trials.

## Methods

### Production cell lines

The amino acid sequence of the 3D7 Pfs25 protein (GenBank^TM^ accession number P13829, formerly AAN35500.1) was used to design a yeast codon-optimized gene (accession number OM525776) corresponding to Lys_23_ to Thr_193_ with the removal of the three N-linked glycosylation sites (NxS/T) at N112, N165, and N187 by mutating Asn to Gln. The Pfs25H expression plasmid was prepared by cloning a SnaBI/SpeI fragment (accession number OM525776) into SnaBI/AvrII digested pPIC9K (Invitrogen). The pPIC9K/Pfs25H plasmid was transformed into *P. pastoris* and a resulting Pfs25H transformant subsequently transformed with pPICZαA/PpPDI to generate the Pfs25H/PDI production clone^3^. The Pfs25M expression plasmid was generated by cloning an XhoI/XbaI (accession number OM525776) fragment into the XhoI/AvrII sites of a modified version of pPIC9K in which the XhoI site at position 5709 had been converted to an XbaI site and contained a copy of the *Pichia* PDI gene under the control of the AOX1 promoter cloned into the AatII site. The synthetic gene contained a Lys-Arg Kex2 cleavage site immediately following the 5’ XhoI cloning site resulting in the secretion of mature Pfs25M protein that contained no heterologous amino acid residues. The PDI co-expression plasmid was linearized with StuI and transformed into *P. pastoris* to generate the Pfs25M/PDI production cell line.

### Recombinant Pfs25M and conjugated Pfs25M-EPA production: fermentation, purification, and conjugation

Production of Pfs25H has been previously described^3^ (see Supplementary Methods for details). Pfs25M was fermented similarly to Pfs25H^3^ with the exception that the methanol induction conditions used were 25 °C, pH 3.5, and methanol feed rate of 375 mL/L/h for 48 to 52 h at a 60 L scale using a New Brunswick BioFlo 3000 fermentation skid. The Pfs25M was harvested by processing through a 750,000 NMWC (GE Healthcare) filter to separate the cells from the supernatant. The filtered supernatant was collected and concentrated ~6X and dialyzed into 20 mM citrate phosphate buffer, pH 6.0. The Pfs25M purification process involves four-column procedures. The Pfs25M protein was diluted into 20 mM citrate phosphate buffer, pH 4.6 (<5 mS/cm conductivity), and was captured using a strong cation exchanger (SP Sepharose FF, GE Healthcare) using a high linear flow rate to process the load quickly. The Pfs25M protein eluted off the column in 20 mM citrate phosphate buffer with 250 mM NaCl. Captured Pfs25M was diluted with 3.6 M ammonium sulfate, pH 7.4 to adjust conductivity to a final concentration of 1.8 M ammonium sulfate. After bulk dilution, the Pfs25M was purified using hydrophobic interaction chromatography on a Phenyl Sepharose HP column (GE Healthcare), equilibrated in 1.8 M ammonium sulfate pH 7.4. This purification step markedly reduces the endotoxin levels. The protein eluted off the Phenyl Sepharose HP in 40 mM Tris-HCl, 0.95 M ammonium sulfate, pH 7.4. The Phenyl Sepharose HP elution product was then dialyzed into 50 mM glycine, pH 9.5 using a 3000 NMWC hollow fiber filter in preparation for loading onto a Q Sepharose FF column. The dialyzed Pfs25M protein was passed through a strong anion exchanger (Q Sepharose FF, GE Healthcare) where the monomer protein passes through, and the dimer protein is captured; during this step host cell protein and endotoxin levels are further reduced. The Q Sepharose FF eluted Pfs25M was concentrated, then polished using size exclusion chromatography (Superdex 75, GE Healthcare) into 1X PBS, pH 7.4. The final purified bulk substance is filtered using a 0.22 µm filter. Details for the process for scaled conjugation are included in Supplementary Methods.

### Protein characterization

Each recombinant protein was characterized using similar techniques to that reported^22,23^ with the following changes. Reversed-phase chromatography was done on a UPLC system with an ACQUITY UPLC Protein BEH C4, 300 Å column (Waters Corp). Analytical size exclusion chromatography (SEC) with in-line multiangle light scattering (MALS) and quasielastic light scattering were performed on a UPLC system with a microDAWN detector (Wyatt Tech). Protein size separation was done on an ACQUITY UPLC Protein BEH SEC 200 Å column (Waters Corp). Amino-terminal protein sequencing was performed by the Research Technologies Branch, NIAID, NIH by Edman degradation on a Sequenator model Procise 494 HT (Applied Biosystems). Electrospray ionization mass spectrometry was done on an automated chip-based nanoelectrospray unit, TriVersa Nanomate (Advion BioSciences; details in Supplementary Methods). Circular dichroism (CD) spectroscopy was performed on a Jasco J-815 spectropolarimeter (details in Supplementary Methods). Endotoxin levels were measured by *Limulus* amoebocyte lysate in a 96-well plate with chromogenic reagents and PyroSoft software (Associates of Cape Cod Inc., East Falmouth, MA) as described by the manufacturer. The host cell protein content was determined by immunizing rats with a *P. pastoris* host cell protein mixture to produce antisera that was used as previously described for *Escherichia coli*^24^ (details in Supplementary Methods).

### Atomic force microscopy of Pfs25M-EPA nanoparticles

For characterization in buffer solution, quantitative nanomechanical (QNM) peak-force AFM experiments were carried out on a multimode 8-nanoscope V instrument (Bruker, CA; details in Supplementary Methods). Herein we imaged under PBS buffer using MSNL cantilevers and calibrated spring constant designed between 0.01 and 0.3 N/m. The peak force setpoint is kept at a selected value between 50 pN and 300 pN for soft nanoparticles. Typically, 10 μl drops of Pfs25M-EPA stock solution prediluted to 1–10 µg/mL range were deposited on a freshly peeled mica surface and allowed to absorb for 3–20 min and then imaged under the same buffer following optimization for biological AFM. For AFM characterization in air at higher resolution, the above samples were rinsed by adding and removing 200 µl purified water 3 to 5 times and dehydrated under a gentle flow of dry nitrogen (details in Supplementary Methods). Tapping-mode imaging in air was carried out on two multimode 8-nanoscope V instruments (Bruker, CA, details in Supplementary Methods). Peak force AFM imaging in air was also carried out on these instruments but with an ultra-sharp silicon tip and low spring constant of 0.12 N/m (PeakForce-HIRS-F-B, Bruker, CA) in its designed high-speed holder. Data were analyzed with instrument-associated software (Nanoscope Analysis ver 2.0, Bruker, CA) and exported as ASCII files for further analysis and display with Fiji/ImageJ (ver 1.53×, NIH, Bethesda, MD) and Excel (Microsoft, Richmond, WA). Nanoparticle shapes were visualized by topological maps and represented by an equivalent diameter, computed from EqDiameter = (6*Volume/π)^(1/3). The mechanical compliance for individual nanoparticles was calculated from Cp = (Deformation – background)/(peak force) at each QNM imaging pixel with topological height above 5 nm and then statistically averaged.

### Generation of Pfs25M and Pfs25H specific antisera

Female BALB/c mice 6–8 weeks of age, or female New Zealand White rabbits 12–14 weeks of age, were immunized thrice with purified recombinant protein formulated in a water-in-oil adjuvant i.e., Montanide ISA720 following 21-day schedule (details in Supplementary Methods) Groups of eight or five *Macaca mulatta* (rhesus) were randomized by age, sex, and weight according to each study protocol. Vaccinations were performed on days 0, 56, and 112 (Pfs25H-EPA) or 168 (Pfs25M-EPA) by intramuscular injection. The non-human primate study used a clinical formulation for Pfs25H-EPA and a research lot for Pfs25M-EPA. Specifically, 47 µg conjugated Pfs25 in 0.6 mL on 1.6 mg/mL Alhydrogel in PBS was used. Sera used in the reported assays were collected 2-weeks post-third immunization and pooled (day 126 for Pfs25H-EPA or day 183 for Pfs25M-EPA). All animals used for this project were approved by the National Institute of Allergy and Infectious Diseases, Division of Intramural Research, Animal Care and Use Committee, protocol: ASP LMIV 1E for small animals and ASP LMIV 9E for NHP at the National Institutes of Health which is AAALAC accredited and OLAW assured. The NIAID DIR Animal Care and Use Program acknowledges and accepts responsibility for the care and use of animals involved in activities covered by the NIH IRP’s PHS Assurance #A4149-01.

### Monoclonal antibody and recombinant antibody production

Pfs25-specific mAbs were expanded in vitro by Precision Antibody™ (Columbia, MD) and secreted IgG was used for Protein G purification as recommended by the manufacturer (Pierce/Thermo Fisher Scientific). Nucleotide sequencing of mAb 1G2 was performed by LakePharma, CA. The accession numbers for the 1G2 H and L chains are OM331740 and OM33174, respectively. Heavy and light chains from the 1G2 hybridoma were amplified and ligated into the pVRC8400 vector (details in Supplementary Methods). One liter of HEK293 FreeStyle cells (Invitrogen catalog number R79007) at 1 × 10^6^ cells/mL in FreeStyle media (Invitrogen catalog number 12338021) were simultaneously transfected with 0.5 mg of heavy and 0.5 mg light chain vector with 1 mL 293fectin (Invitrogen catalog number 12347019) according to the manufacturers’ protocol to produce recombinant antibodies. After 120 h, the cell medium supernatant containing antibody was loaded onto a 5 ml Protein A column. Following 20 column volumes of wash with 20 mM Tris with 50 mM sodium chloride pH 6.8, tobacco etch virus (TEV) protease (Sigma Aldrich part number 4455-10KU) was added to the Protein A column at 0.125 times the mass of bound antibody and incubated overnight at 4 °C. After washing with 5 column volumes of wash buffer, the flow through was concentrated to 1 ml and passed through a nickel spin column to remove the his-tagged TEV protease. Fab was then incubated with a two-fold excess of Pfs25M. The Pfs25M/Fab complex was loaded onto a Hi-load 16/60 prep grade Superdex 75 size exclusion column (GE part number 17-1068-01). Fractions were recovered and the complex was identified by SDS-PAGE. Pooled fractions were concentrated using 0.5 ml centrifugal filters (Amicon Ultra part number UFC500396).

### Western blotting, mAb capture ELISAs, and competition ELISAs

Western blots with Pfs25M or Pfs25H specific mAbs 4B7, 1G2, and 4F7 were performed using each mAb at ~5 µg/mL. Recombinant Pfs25H or Pfs25M were loaded (1 µg per lane) onto a 4–20% tris-glycine gel (Invitrogen Cat. # XP04202BOX) under non-reduced or reduced and alkylated conditions. Reduction was performed at 15 mM dithiothreitol and 60 °C for 30 min and alkylation was performed at 70 mM iodoacetamide and room temperature in the dark for 30 min. The gel was transferred onto a nitrocellulose membrane and probed with individual mAbs followed by the secondary antibody goat anti-mouse IgG (H + L) antibody human serum adsorbed (Seracare Cat # 5220-0357). Detection was done with BCIP/NBT phosphatase substrate (Seracare Cat. #5420-0038).

ELISAs to assess rhesus antibody responses were performed as described (7) using goat anti-human IgG (H + L) peroxidase-labeled (Seracare Cat.# 5220-0330). To evaluate Pfs25M and Pfs25H antibody binding in solution by mAbs 4B7 and 1G2, each mAb was plated at 5 µg/mL in antibody buffered solution for 2 h, washed, blocked, and incubated with 5 µg/mL Pfs25M or Pfs25H. Wells were washed, incubated with diluted Pfs25M specific rabbit antisera and bound rabbit IgG was detected with goat anti-rabbit IgG HRP labeled at a 1:1000 dilution (Seracare Cat.# 5220-0336). The competition ELISAs were performed by plating Pfs25M as capture antigen, and the murine mAbs 1G2 or 4B7, following direct coupling with HRP as per the manufacturer’s guidelines (EZ-link™ Plus Activated Peroxidase Kit, ThermoFisher Scientific), were maintained at a constant dilution while the purified rhesus IgG was titrated in antibody binding buffer. The plates were incubated for 60 min then washed 4X and incubated with HRP substrate (TMB Chromogen Solution, ThermoFisher Scientific).

### Assessment of transmission-blocking activity

The standard membrane feeding assay (SMFA) assessing the blocking of parasite transmission of *P. falciparum* NF54 parasites cultured in vitro and membrane feeding assay were performed (details in Supplementary Methods). Briefly, test samples (either serum, or total IgG) were diluted and mixed with a gametocyte culture of *P. falciparum* (NF54 strain). The mixture was fed to *Anopheles stephensi* (Nijmegen strain) mosquitoes through a membrane-feeding apparatus. Mosquitoes were kept for 7–8 days and dissected to enumerate the oocysts in the midgut.

### Isothermal titration calorimetry

Pfs25M, Pfs25H, and 1G2 IgG solutions were prepared for isothermal titration calorimetry (ITC) experiments by dialysis against PBS for 2 h. Prior to each experiment, the Pfs25M, Pfs25H, and 1G2 protein solutions were filtered through a Whatman inorganic membrane filter or 0.22 µm filter, checked for the presence of aggregates by dynamic light scattering using a Malvern Nano-S instrument, and degassed for 30 min using the TA Instruments degassing station. The solutions containing Pfs25M or Pfs25H were loaded in the syringe at 165 μM or 49 μM, and 1G2 was placed in the calorimeter cell at 23 μM or 3 μM, respectively. Binding experiments were performed on a TA Instruments Low Volume Nano ITC instrument at 25 °C using 20 injections of 2.5 μL each, with injection interval of 300 s, and a syringe stirring speed of 250 rpm. The initial injections were excluded from the data analyses. The ITC data were fitted to the independent binding site model using the TA Instruments NanoAnalyze software.

### Pfs25M-1G2 Fab structure determination

Native polyacrylamide gels were used to titrate the amount of each protein needed to form a 1:1 complex between Pfs25M and the 1G2 Fab. Protein crystals of the complex were prepared by mixing 1 µl of Pfs25M-1G2 Fab complex with 1 µl of the crystallization solution consisting of 0.1 M Hepes pH 7.5 and 20–24% polyethylene glycol 6000, and 20% glycerol with and without the additives 1,6-hexanediol and 1,3-propanediol in a hanging drop experiment. Better diffracting crystals were obtained with additives which were flash-frozen and X-ray data were collected at 1.00 Å at beamline 22-ID at Argonne National Laboratory. Data were processed using XDS (Supplementary Table 1)^25^. Molecular replacement using in-house antibody models allowed a preliminary trace of Pfs25M. Iterative model building with Coot^26^ and refinement with Phenix^27^ yielded excellent electron density (Supplementary Fig. 10) and allowed the tracing of the two N-terminal domains of Pfs25M. Coordinates and data for the Pfs25M-1G2 complex are deposited at the RCSB with accession code: 7TXW.

### Reporting summary

Further information on research design is available in the Nature Research Reporting Summary linked to this article.

## Supplementary information

Supplementary Material

REPORTING SUMMARY

## Data Availability

The datasets generated during and/or analyzed during the current study are available from the corresponding author upon reasonable request. Coordinates and X-ray data for the Pfs25M-1G2 complex are deposited at the RCSB with accession code: 7TXW.
