# Supplementary material for: Structural and immunological differences in Plasmodium falciparum sexual stage transmission-blocking vaccines comprised of Pfs25-EPA nanoparticles
Source: NPJ Vaccines. 2023 Apr 15;8:56. doi: 10.1038/s41541-023-00655-5 (PMC10105769; doi:10.1038/s41541-023-00655-5)
Supplement: Supplementary file 1 — Supplementary Material [file 41541_2023_655_MOESM1_ESM.docx]

**Supplementary Material**

**Structural and immunological differences in *Plasmodium falciparum* sexual stage transmission-blocking vaccines comprised of Pfs25-EPA nanoparticles**

**Authors:**

Nicholas J. MacDonald, Kavita Singh, Karine Reiter, Vu Nguyen, Richard Shimp, Jr., Apostolos G. Gittis, Beth Chen, Marty Burkhardt, Baoshan Zhang, Zhixiong Wang, Raul Herrera, Mackenzie Moler, Duck-Yeon Lee, Sachy Orr-Gonzalez, Jessica Herrod, Lynn Lambert, Kelly M. Rausch, Olga Muratova, David S. Jones, Yimin Wu, Albert J. Jin, David N. Garboczi, Patrick E. Duffy and David L. Narum

**Included:**

Supplementary Methods

Supplementary References

Supplementary Table 1

Supplementary Figures 1-10

# SUPPLEMENTARY METHODS

## Production of Pfs25-PpPDI and Pfs25-PfPDI in 5 L bioreactors and harvest

Selected Pfs25-PpPDI and Pfs25-PfPDI clones were tested in 5 L bioreactors, using a New Brunswick BioFlo3000 Mobile Pilot Plant (New Brunswick Scientific, New Brunswick, NJ). The cultivation has been described previously^1^ except for the temperature during induction was lowered to 25 °C. The pH and temperature were reduced at this phase to 3.5 and 25 °C, respectively. Samples were taken and analyzed every 4 h by SDS-PAGE throughout the methanol induction phase. Fermentation broth was processed for downstream purification using centrifugation, or microfiltration and ultrafiltration as described.^1^ The clarified fermentation broth was diafiltered against 2 × PBS pH 7.4 and stored at −80 °C until tested.

## Purification of recombinant Pfs25

Recombinant Pfs25 was purified following the procedure described earlier with some modifications.^1^ Briefly, UF/DF material from fermentation supernatant was captured on a nickel nitrilo-triacetic acid Superflow (Ni-NTA) column (Qiagen Inc., Valencia, CA) and captured material was eluted as described. The elution pool was titrated to a final of 1.8 M ammonium sulfate using 3.5 M ammonium sulfate and loaded on a phenyl Sepharose HP (GE Healthcare, Piscataway, NJ) hydrophobic interaction chromatography column (4 mg Pfs25/mL resin) pre-equilibrated with ammonium sulfate buffer. Pfs25(A) and Pfs25(B) was eluted by step elution with 1.2 M and 0.5 M ammonium sulfate, respectively. The elution pool was loaded on a Superdex 75 size-exclusion column (Amersham Bioscience) equilibrated with saline. The load for the size-exclusion column ranged from 5 to 6% of one column volume. Purified recombinant Pfs25(A) was biochemically characterized as described below.

# Process for Scaled conjugation

Process development of Pfs25-EPA conjugates followed these procedures previously reported^2^: Initial development used scaled-down procedures based on preset pilot-scale conditions to determine the best conditions for linker modification of Pfs25H and EPA, as well as conditions for forming the protein–protein conjugates. The thiol-modified Pfs25H (Pfs25-SH) and maleimides-modified EPA (EPA-mal) were prepared using commercially available reagents SATA and EMCS (Pierce Biotech, Rockford, IL), respectively. The detailed materials and methods for pilot-scale development are included below:

## Dialysis, and concentration and modification of Pfs25H and EPA

The Pfs25H protein was concentrated and dialyzed into 100 mM Sodium Phosphate, 154 mM NaCl, 5 mM EDTA, pH 7.3 using a size 5, 3K nominal molecular weight cutoff (NMWC) hollow fiber filter (GE Healthcare, Piscataway, NJ) with pressure maintained at ≤ 25 PSIG and then concentrated to 2.25 ± 0.25 mg/mL based on the A280. The dialyzed Pfs25H was chemically modified by adding a 100 mM solution of N-Succinimidyl S-Acetylthioacetate (SATA, Pierce Biotech, Rockford, IL) in DMSO at 22 ± 5°C. SATA was added to the Pfs25H dialyzed protein at 5 x 10^-7^ moles/mg of Pfs25H and mixed at 300 ± 25 RPM at 22 ± 3°C for 60 ± 5 min. After mixing, the dialysis step was repeated as described above with the same buffer to remove the free SATA and DMSO followed by concentration to 2.25 ± 0.25 mg/mL as determined by A280.

The EPA stock protein solution was dialyzed into 100 mM Sodium Phosphate, 154 mM NaCl, 5 mM EDTA, pH 7.3 and then concentrated to 3.0 ± 0.25 mg/mL as determined by A280 using a 10 K NMWC hollow fiber filter (GE Healthcare, Piscataway, NJ). The EPA protein was modified by addition of a 100 mM solution of N-(ε-maleimidocaproyloxy)-succinimide ester (EMCS, Pierce Biotech, Rockford, IL) in DMSO at 6.73 x 10^-7^ moles/mg of EPA and mixed at 300 ± 25 RPM at 22 ± 3°C for 90 ± 5 min. During protein cross linking, the 3 K and 10 K NMWC hollow fiber filters were regenerated and equilibrated in 100 mM Sodium Phosphate, 154 mM Sodium Chloride, 5 mM EDTA, pH 6.5.

The concentration of thiol linkers was determined colorimetrically using 4,4’-dithiodipyridine as described ^3^. Maleimide linkers were determined indirectly by mixing with a known concentration of cysteine and measuring cysteine thiol consumption.

## Deacetylation

Pfs25-SATA was deacetylated by adding a 10% v/v of a 10X stock solution (96 mM Sodium Phosphate, 154 mM Sodium Chloride, 25 mM EDTA, 500 mM hydroxylamine HCl, pH 7.3) and mixing for 60 ± 5 min at 300 ± 25 RPM at 22 ± 3 °C.

## Dialysis of Pfs25-SH and EPA-EMCS

After deacetylation, Pfs25-SH protein was dialyzed in100 mM Sodium Phosphate, 154 mM Sodium Chloride, 5 mM EDTA, pH 6.5 and the concentration was adjusted to 5 mg/mL based on A280. Within the same period of time, after EPA was modified with EMCS (EPA-EMCS), EPA-EMCS was dialyzed into the buffer as above and concentration was adjusted to 5 mg/mL based on A280.

## Conjugation of Pfs25-SH to EPA-EMCS

The conjugation step required approximately three moles of Pfs25-SH to each mole of EPA-EMCS. The EPA-EMCS was added to the Pfs25-SH and mixed at 300 ± 25 rpm, 22 ± 3°C for 1 hr. The conjugation reaction was quenched by adding 57 mM L-Cysteine HCl to make a final concentration of 0.38 mM L-Cysteine HCl.

## Post conjugation purification and bulk fill

The Pfs25-EPA conjugate (5% v/v) was applied to a Sephacryl S-300 column (GE Healthcare) with a total bed height of 60 cm equilibrated with 154 mM NaCl, 2.97 mM Sodium Phosphate, 1.04 mM Potassium Phosphate, pH 7.4 (SEC buffer) at 25 cm/hr.

The S-300 product peak which comprised approximately 70% of the column eluate was pooled based on the retention time and the A280 profile and concentrated using a 300,000 NMWC hollow fiber filter (GE Healthcare, Piscataway, NJ) equilibrated with SEC buffer. The S-400 elution pool was concentrated until the A280 was between 0.9 – 1.1 AU, filter sterilized using a 0.22 µm filter cartridge, aliquoted in PETG bottles and stored at < -70°C.

# **Electrospray ionization mass spectrometry (ESI-MS)**

ESI-MS was done on an automated chip-based nanoelectrospray unit, TriVersa Nanomate (Advion BioSciences) as previously described^4^: Intact mass analyses were performed by electrospray ionization mass spectrometry (ESI-MS). Samples were analyzed by coupling an automated chip-based nanoelectrospray unit, TriVersa Nanomate (Advion BioSciences), to a 4000 Q Trap liquid chromatography-tandem MS (LC-MS/MS) device (Applied Biosystems/Sciex). Sample solutions were first adjusted to low pH (0.1% acetic acid) and low acetonitrile (2%) and then desalted through a microprotein trap cartridge (Michrom BioResources) at 20 ml/min. In LC-MS mode, a Tempo multidimensional liquid chromatography (MDLC) device designed for HPLC applications was used to automate trap desalting of protein samples. Bound protein was step eluted with 60% acetonitrile and 0.1% acetic acid at 800 nl/min into the 4000 Q Trap via a TriVersa capillary coupler.

# Circular dichroism (CD) spectroscopy

CD was performed on a Jasco J-815 spectropolarimeter as previously described^5^: To examine secondary structure, samples in 1× PBS were diluted 1:10 in deionized (MilliQ) water. CD spectra were recorded over the wavelength range 185–260 nm in a 1-mm path length quartz cuvette using a step size of 0.2 nm, a slit bandwidth of 1.0 nm, and a signal averaging time of 1.0 s. Analysis of temperature on secondary structure was performed in 5 °C temperature increments from 5 to 80 °C. Secondary structure content was calculated using the DICHROWEB web server.

# **Analytical chromatography**

## SEC-HPLC-MALS

The Pfs25 conjugates were analyzed by size-exclusion chromatography with in-line multi-angle light scattering as previously described.^6^ Samples were run on a TSKgel G4000SWxl column (Tosoh Bioscience) with an isocratic gradient of PBS + 308 mM NaCl at a flow rate of 0.5 mL/min.

## RP-HPLC

The Pfs25 conjugates were analyzed by reversed-phase chromatography. Briefly, samples were run on a POROS R2 10 µm, 2.1 x 30 mm column. The initial mobile phase combined 95% mobile phase A (0.1% (w/v) TFA in water) and 5% mobile phase B (0.1% (w/v) TFA in acetonitrile) and the conjugate was eluted by increasing the mobile phase B to 100% over 38 minutes at a flow rate of 1 mL/min.

# **Host cell protein content**

Host cell protein content (HCP) was determined using a method developed for *E. coli*^7^ by immunizing rats with a *P. pastoris* host cell protein mixture derived from an overexpressing PDI null clone that was fermented in a 5L bioreactor following the procedures developed for Pfs25M with PDI and recovered by centrifugation. Frozen cell pellets were ^8^ All animal work was performed in accordance with National Institutes of Health guidelines and under the auspices of an Animal Care and Use Committee approved protocol.

For slot blotting, HCP standard dilutions ranged from approximately 3 to 1500 ng/mL, while the purified protein dilutions ranged from 50 to 100,000 ng/mL. To control for potential interference between HCPs and the purified proteins, serial dilutions of both were mixed and loaded in a separate lane (in amounts equivalent to the respective loads in the other lanes). The slot blot membrane was washed then incubated with pooled antisera at a dilution of 1:1000 at room temperature for 1 h. After extensive washing with TBS/T, the membranes were incubated at room temperature for 1 h with goat anti-rat IgG-alkaline phosphatase conjugate (reconstituted according to manufacturer's instructions, then diluted 1:3000 in blocking buffer). Following further washes, the membranes were incubated in BCIP/NBT substrate according to manufacturer's instructions for 40 min to allow color development. The membranes were then scanned with a laser densitometer (Molecular Dynamics Personal Densitometer SI, Molecular Dynamics, Sunnyvale, CA) and the scanned images were analyzed by ImageQuant software version 5.2 (Molecular Dynamics).

# Atomic force microscopy of Pfs25M-EPA nanoparticles

For characterization in buffer solution, quantitative nanomechanical (QNM) peak-force AFM experiments were carried out on a multimode 8-nanoscope V instrument (Bruker, CA) as described previously^9^: QNM peak force AFM experiments were carried out on a multimode 8-nanoscope V instrument (Bruker, CA) under pH 6.0 buffer using an MSCT cantilever with its calibrated spring constant designed between 0.01 to 0.03 N/m and peak force setpoints of 100–200 pN; 5 μl drops of fresh cages +/− auxilin, ATP and Hsc70 or Hsc70ΔC were deposited on freshly peeled mica and imaged under the same buffer following routine optimization for biological AFM. Data were analyzed with instrument software (Nanoscope ver8.15, Bruker, CA) and exported as ascii files for further analysis with Excel (Microsoft, Richmond, WA), and displayed with ImageJ (ver 1.4x, NIH, Bethesda, MD). Samples as described for AFM were absorbed for 20 seconds to a Formvar-carbon grid, followed by a rinse and 20 second incubation with 1% uranyl acetate. Grids were wicked, air dried, and examined at 25,000× magnification in a JEOL JEM 1200EX-II.

For AFM characterization in air at higher resolution, the above samples were rinsed by adding and removing 200 µl purified water 3 to 5 times and dehydrated under a gentle flow of dry nitrogen, as previously described^2^: Biological imaging was carried out under a range of conditions, both in fluid and air, using gentle tapping-mode AFM, mostly with a PicoForce Multimode AFM (Bruker, CA) consisting of a Nanoscope® V controller, a type E scanner head, and a sharpened TESP-SS (Bruker, CA) or similar AFM cantilever ^10,11^. For Pfs25-EPA visualization, suitable protein attachment was readily achieved by a ten-minute incubation of 7 μl of the sample solution diluted to about 1.7 nM in PBS (pH = 7.4) buffer on freshly peeled mica substrates, followed by rinsing with c.a. 1 ml of deionized water and complete drying under an inert gas flow. The sample was then sealed into the instrument compartment dehumidified by Drierite® particles ^5,10^. AFM images were evaluated within the Nanoscope software (version 7.3 to 8.1, Bruker, CA), and exported to Image J (version 1.41o, NIH, Bethesda, MD) and Mathcad (version 14, Mathsoft, MA) for further analyses and display.

# Generation of Pfs25M and Pfs25H specific antisera

Pfs25M- and Pfs25H-specific antisera were generated as previously described^12^: Paired New Zealand White rabbits were immunized three times (days: 0, 28, and 56) with 50 μg of purified Pfs25M or Pfs25H formulated in ISA 720 VG (Seppic, Inc., Fairfield, NJ) administered subcutaneously. Rabbits were bled for sera on day 0 and 2 weeks following their third immunization for subsequent testing. Rabbit IgG purification used Protein G column chromatography as suggested by the manufacturer (GE Healthcare). All IgG samples were dialyzed extensively in PBS, pH 7.4.

**Monoclonal antibody and recombinant antibody production**

Pfs25-specific mAbs were expanded *in vitro* by Precision Antibody™ (Columbia, MD) and secreted IgG were used for Protein G purification as recommended by the manufacturer (Pierce/Thermo Fisher Scientific). Nucleotide sequencing of mAb 1G2 was performed by LakePharma, CA. The accession numbers for the 1G2 H and L chains are OM331740 and OM33174, respectively. Heavy and light chains from the 1G2 hybridoma were amplified and ligated into the pVRC8400 vector as previously described^13^: A gene encoding the 1G2 heavy chain with a tobacco etch virus (TEV) protease recognition site (ENLYFQG) inserted after Gly 238 was synthesized and cloned into pVRC8400. Similarly, the IG2 light chain was synthesized and cloned into the pVRC8400 vector. The 1G2 heavy and light chain plasmids were transiently co-transfected into HEK293 FreeStyle cells (Invitrogen R79007) cells, and IgG was purified from the supernatant after 5 days using Protein A agarose (Pierce).

**Standard membrane feeding assay (SMFA)**

SMFA assessing the blocking of parasite transmission of *P. falciparum* NF54 parasites cultured *in vitro* and membrane feeding assay were performed as described^14^: test samples (either serum, total IgG or Pfs25-specific IgG) from animals and humans were diluted and mixed with a gametocyte culture of *P. falciparum* (NF54 strain). The mixture was fed to *Anopheles stephensi* (Nijmegen strain) mosquitoes through a membrane feeding apparatus. Mosquitoes were kept for 8 days and dissected to enumerate the oocysts in the midgut.

# SUPPLEMENTARY REFERENCES

1 Zou, L., Miles, A. P., Wang, J. & Stowers, A. W. Expression of malaria transmission-blocking vaccine antigen Pfs25 in Pichia pastoris for use in human clinical trials. *Vaccine* **21**, 1650-1657, doi:10.1016/s0264-410x(02)00701-6 (2003).

2 Shimp, R. L., Jr. *et al.* Development of a Pfs25-EPA malaria transmission blocking vaccine as a chemically conjugated nanoparticle. *Vaccine* **31**, 2954-2962, doi:10.1016/j.vaccine.2013.04.034 (2013).

3 Riener, C. K., Kada, G. & Gruber, H. J. Quick measurement of protein sulfhydryls with Ellman's reagent and with 4,4'-dithiodipyridine. *Analytical and bioanalytical chemistry* **373**, 266-276, doi:10.1007/s00216-002-1347-2 (2002).

4 Uchime, O. *et al.* Analysis of the conformation and function of the Plasmodium falciparum merozoite proteins MTRAP and PTRAMP. *Eukaryotic cell* **11**, 615-625, doi:10.1128/EC.00039-12 (2012).

5 Plassmeyer, M. L. *et al.* Structure of the Plasmodium falciparum circumsporozoite protein, a leading malaria vaccine candidate. *The Journal of biological chemistry* **284**, 26951-26963, doi:10.1074/jbc.M109.013706 (2009).

6 Jones, D. S. *et al.* A Method for Producing Protein Nanoparticles with Applications in Vaccines. *PLoS One* **11**, e0138761, doi:10.1371/journal.pone.0138761 (2016).

7 Zhu, D., Saul, A. J. & Miles, A. P. A quantitative slot blot assay for host cell protein impurities in recombinant proteins expressed in E. coli. *J Immunol Methods* **306**, 40-50, doi:10.1016/j.jim.2005.07.021 (2005).

8 Singh, S. *et al.* Biochemical and immunological characterization of bacterially expressed and refolded Plasmodium falciparum 42-kilodalton C-terminal merozoite surface protein 1. *Infection and immunity* **71**, 6766-6774, doi:10.1128/IAI.71.12.6766-6774.2003 (2003).

9 Sousa, R. *et al.* Clathrin-coat disassembly illuminates the mechanisms of Hsp70 force generation. *Nat Struct Mol Biol* **23**, 821-829, doi:10.1038/nsmb.3272 (2016).

10 Tsai, C. W. *et al.* Characterization of a protective Escherichia coli-expressed Plasmodium falciparum merozoite surface protein 3 indicates a non-linear, multi-domain structure. *Molecular and biochemical parasitology* **164**, 45-56, doi:10.1016/j.molbiopara.2008.11.006 (2009).

11 Kotova, S. *et al.* AFM visualization of clathrin triskelia under fluid and in air. *FEBS letters* **584**, 44-48, doi:10.1016/j.febslet.2009.11.039 (2010).

12 MacDonald, N. J. *et al.* Structural and Immunological Characterization of Recombinant 6-Cysteine Domains of the Plasmodium falciparum Sexual Stage Protein Pfs230. *The Journal of biological chemistry* **291**, 19913-19922, doi:10.1074/jbc.M116.732305 (2016).

13 McLellan, J. S. *et al.* Structure of HIV-1 gp120 V1/V2 domain with broadly neutralizing antibody PG9. *Nature* **480**, 336-343, doi:10.1038/nature10696 (2011).

14 Cheru, L. *et al.* The IC(50) of anti-Pfs25 antibody in membrane-feeding assay varies among species. *Vaccine* **28**, 4423-4429, doi:10.1016/j.vaccine.2010.04.036 (2010).

# SUPPLEMENTARY TABLES

**Supplementary Table 1.** **X-ray data collection and refinement statistics**

|  | Pfs25M-1G2 complex |
| --- | --- |
| **Data collection** |  |
| Space group | *C*2 |
| Cell dimensions |  |
| *a*, *b*, *c* (Å) | 122.3, 50.2, 100.03 |
| α, β, γ (°) | 90.00, 123.10, 90.00 |
| Resolution (Å) | 61.0-2.17(2.23-2.17) * |
| *R*_merge_ | 6.8(31.8) |
| *I* / σ*I* | 18.62(4.22) |
| Completeness (%) | 98.3(83.6) |
| Redundancy | 4.92(3.46) |
|  |  |
| **Refinement** |  |
| Resolution (Å) | 61.0-2.17(2.23-2.17) |
| No. reflections | 25926 |
| *R*_work_ / *R*_free_ | 17.67/22.99(19.24/26.96) |
| No. atoms |  |
| Protein | 3895 |
| Ligand/ion | 77 |
| Water | 256 |
| *B*-factors |  |
| Protein | 26.32 |
| Ligand/ion | 30.18 |
| Water | 27.66 |
| R.m.s. deviations |  |
| Bond lengths (Å) | 0.010 |
| Bond angles (°) | 1.281 |

A single crystal was used for the structure.

*Values in parentheses are for highest-resolution shell.

# SUPPLEMENTARY FIGURES

B C D E


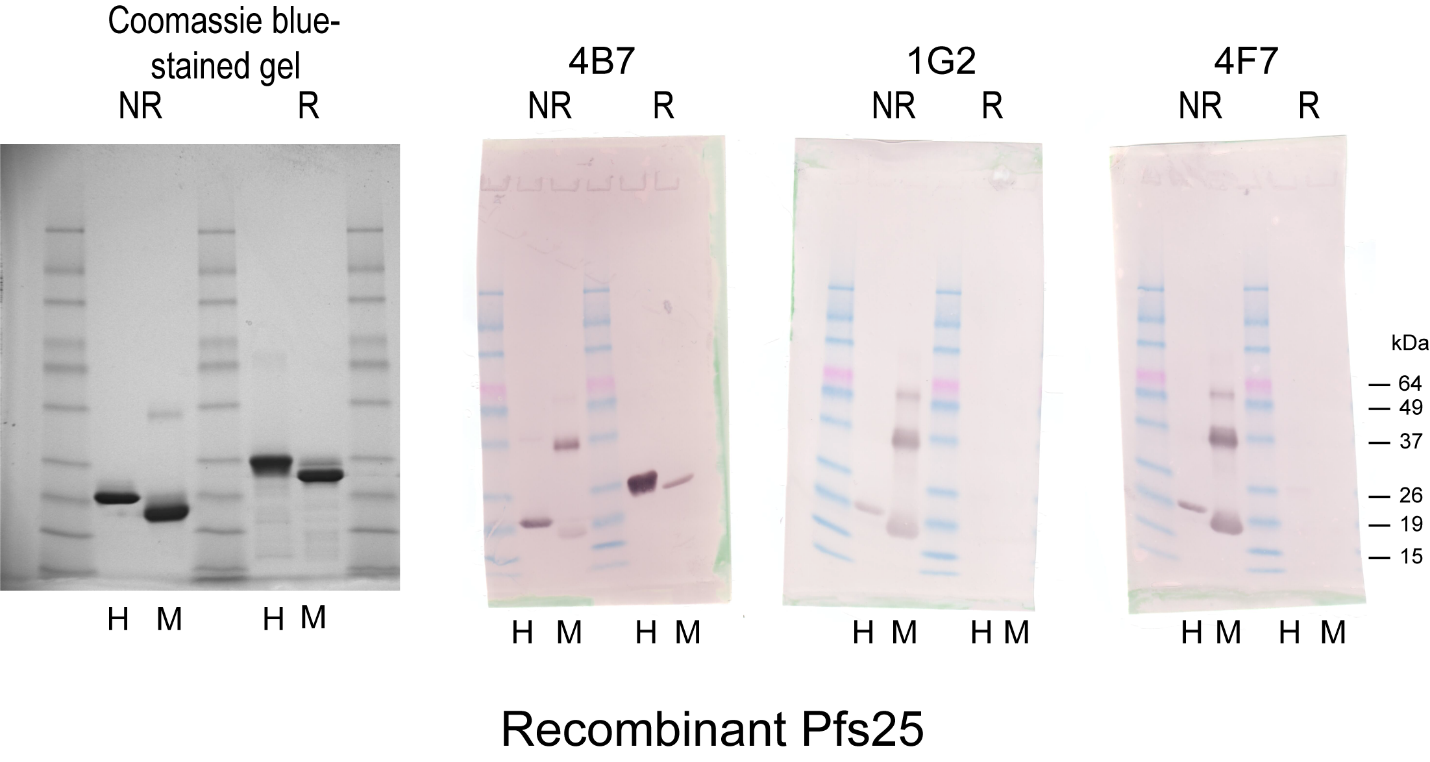


**Supplementary Figure 1. Raw images for edited Figure 1B through E.**

**
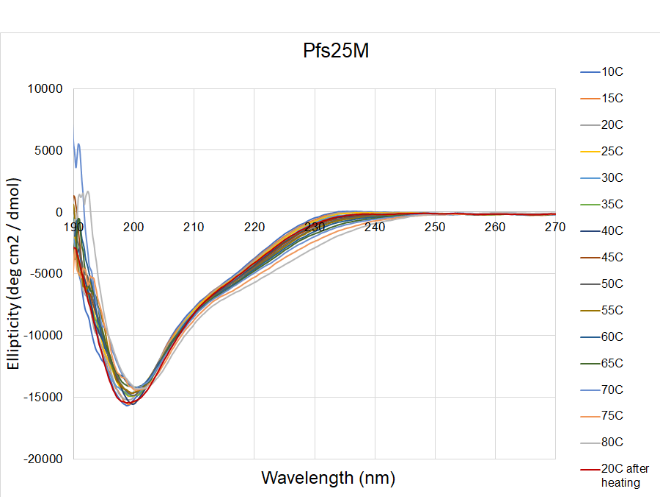

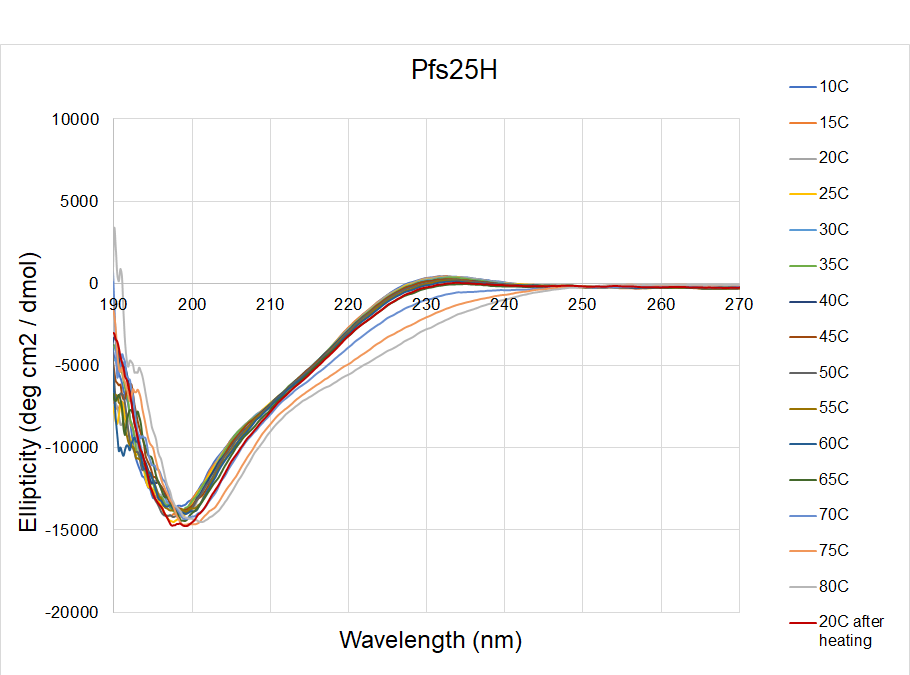
**

Supplementary Figure 2. Thermal stability of Pfs25M compared to Pfs25H by circular dichroism spectroscopy. Thermal stability was assessed using an incremental temperature ramp of 5°C steps.


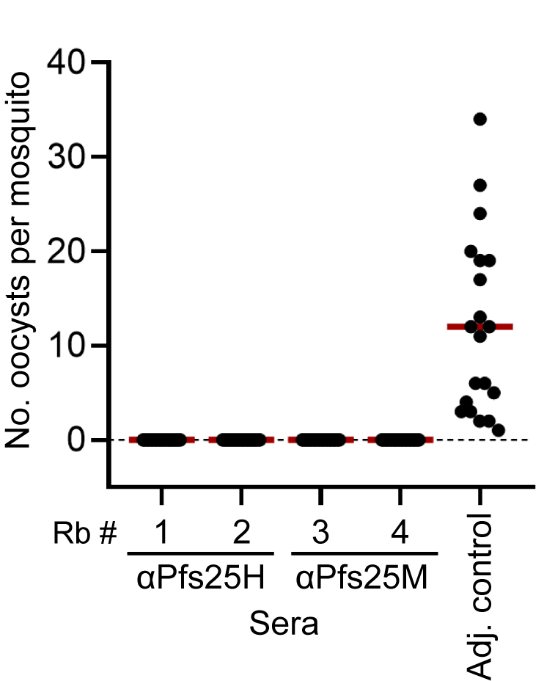


**Supplementary Figure 3. Evaluation of the TRA of rabbit antisera raised against Pfs25H or Pfs25M compared to an adjuvant control using the SMFA.** Sera were heat inactivated, diluted 1:4 and supplemented with human sera containing active complement. Shown are the number of oocysts in a minimum of 20 mosquitoes per sample (●) and the average number of oocysts (**—**). The inhibition of oocyst development was 100% in all test samples compared to the control of 0%.


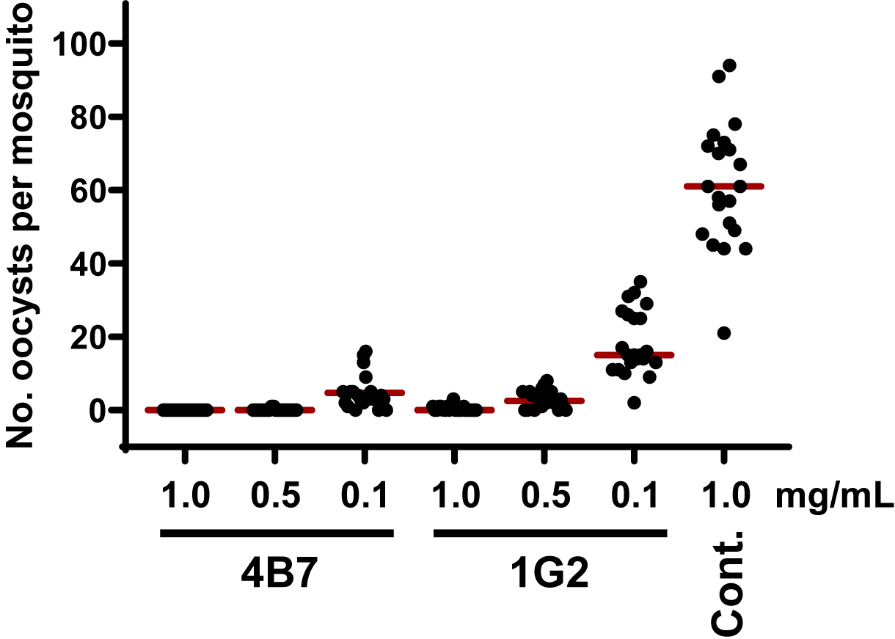


**Supplementary Figure 4. Assessment of TRA of two Pfs25-specific mAbs following a titration against sexual stage parasites.** The average inhibitions of oocyst density following titration of 4B7 or 1G2 from 1.0 to 0.1 mg/mL were 100%, 99.9%, and 92.3%, or 99.3%, 95.3%, and 69.9% using a non Pfs25 specific mAb as a negative control. All assays included human complement.


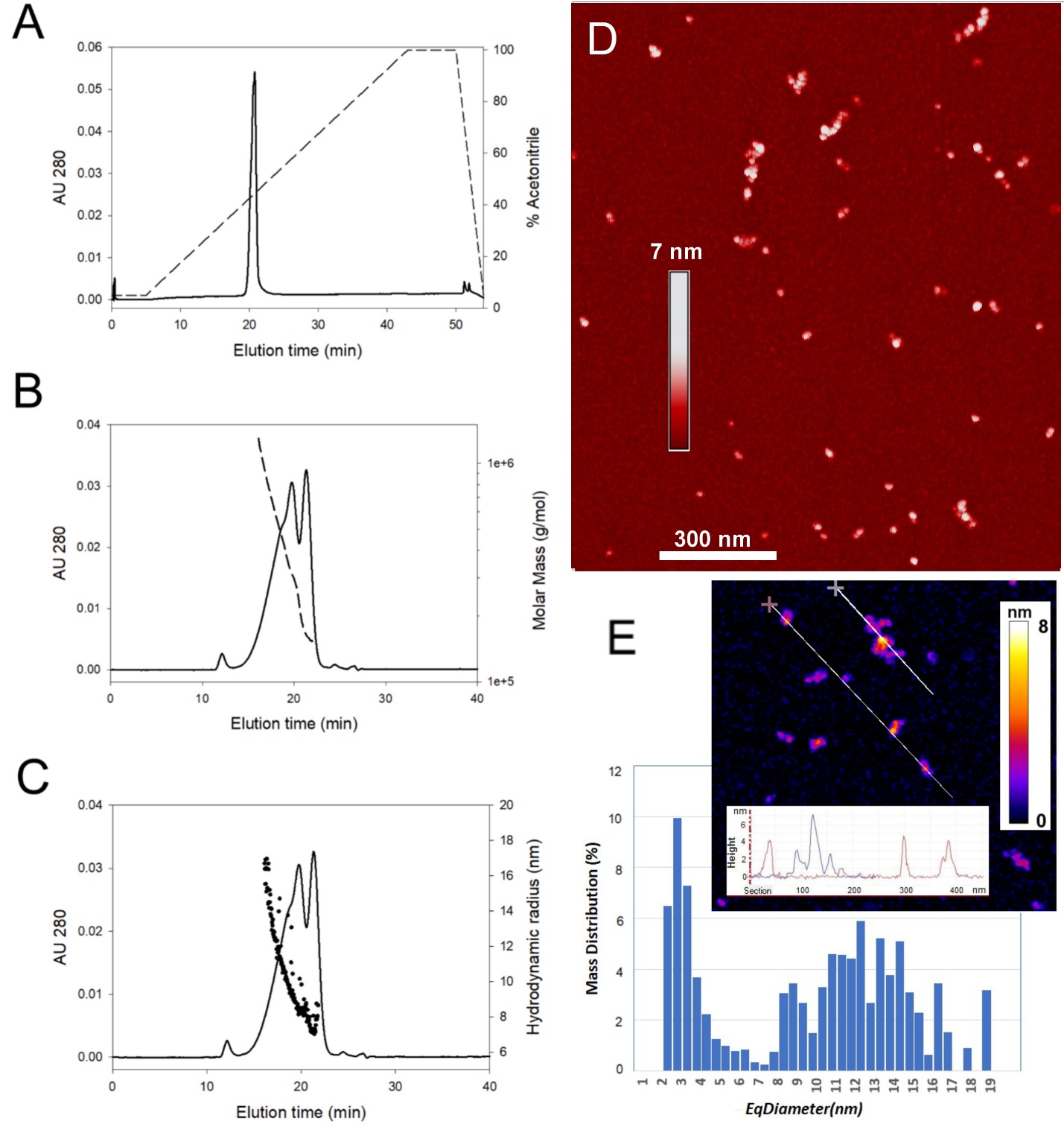


**Supplementary Figure 5. Biochemical and biophysical characterization of conjugated Pfs25M-EPA nanoparticles.** Evaluation of purified recombinant Pfs25M-EPA under non-reduced conditions by reversed-phase HPLC (A) and by analytical SEC-MALS (B), and SEC with in-line QELS (C). High-resolution characterization of conjugated Pfs25M-EPA nanoparticles and their internal constructs by desalting and dehydration and in-air atomic force microscopy on mica with their topological shapes (D) and quantified mass distribution (~370 nanoparticles larger than 5 nm) with a representative insert for height sections (E).


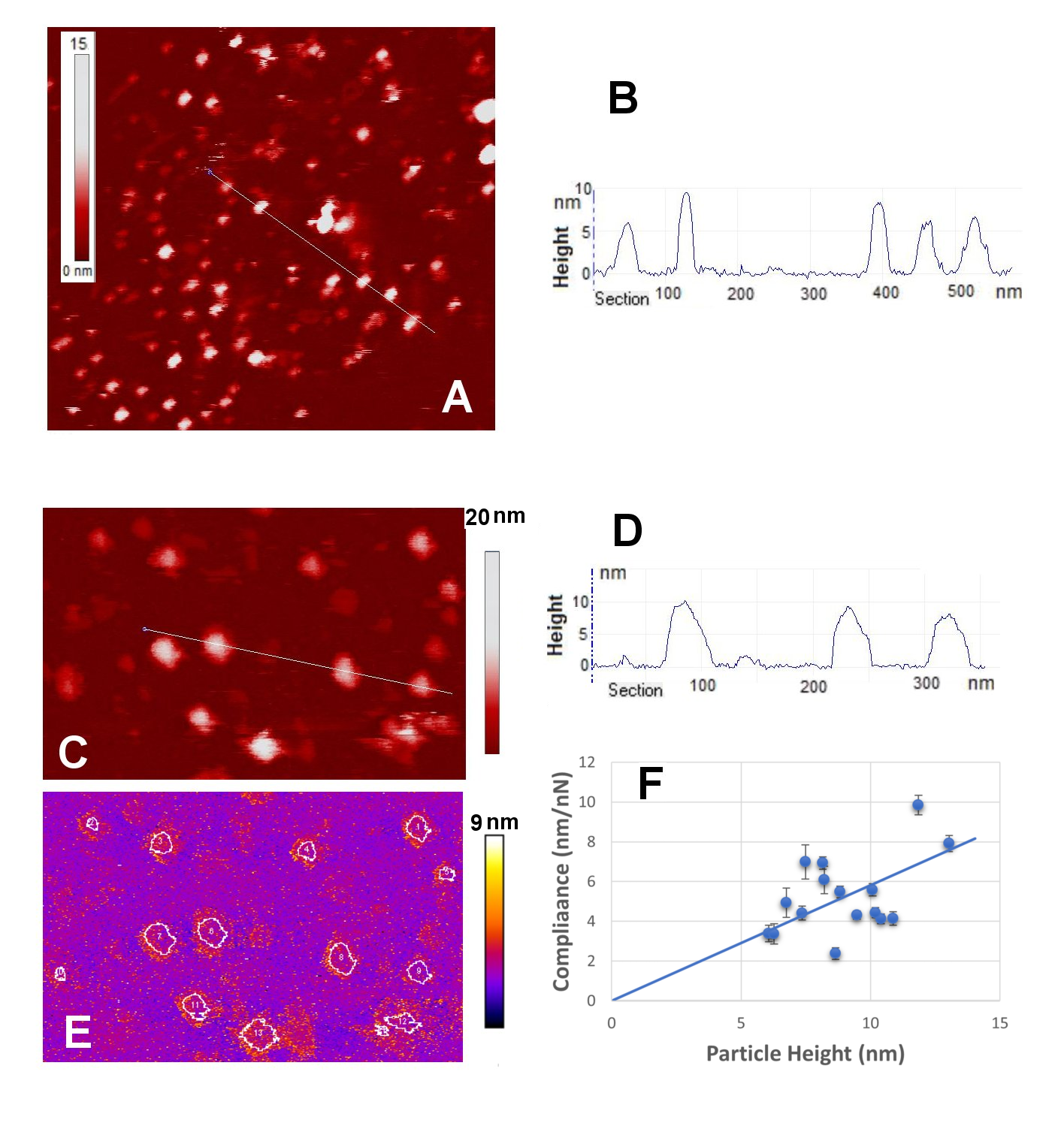


**Supplementary Figure 6.** **In-fluid characterization of conjugated Pfs25M-EPA nanoparticles and their mechanical compliance by quantitative nanomechanical (QNM) atomic force microscopy.** Topological maps (A and C) of the nanoparticle shapes imaged under PBS on mica with representative height sections (B and D). High-resolution deformation measurement (E) of topological map in C shows the mechanical softness of the nanoparticles delineated by their corresponding height topology (C). The calculated compliance (mean +/- standard error of mean) shows both size and nanoscale structural dependency with an average of about 5 nm/nN (F).


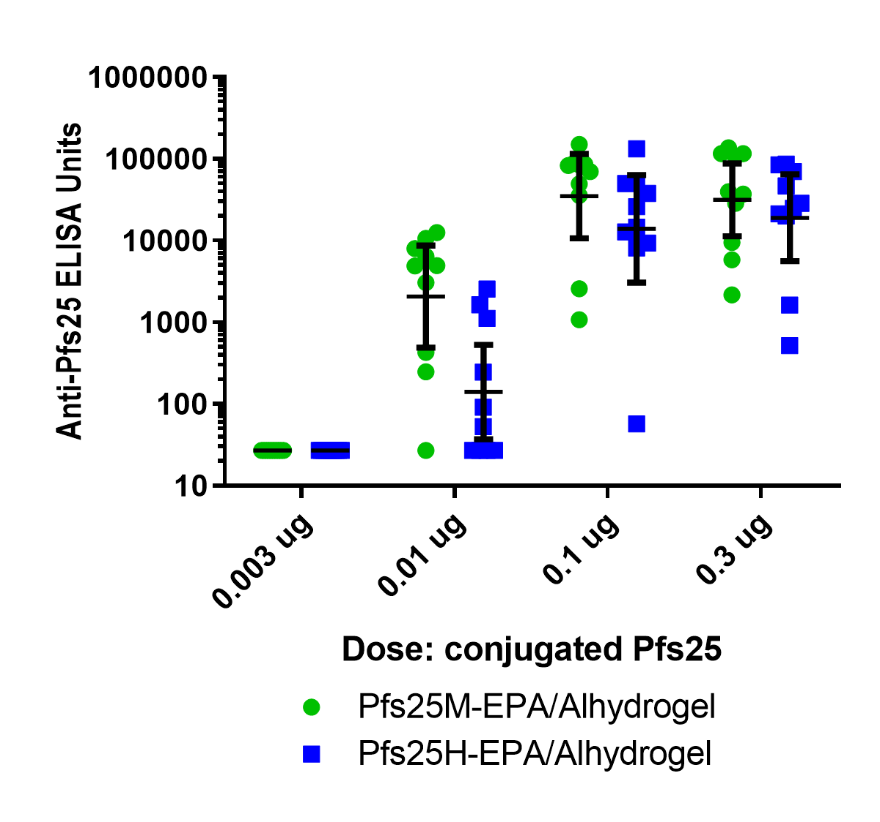


**Supplementary Figure 7. Comparative immunogenicity in mice of Pfs25M-EPA and Pfs25H-EPA conjugated nanoparticles formulated with Alhydrogel.** Mice were immunized with the stated doses on days 0 and 21. Sera were collected on day 35 for ELISAs. The ELISA titers for groups dosed with 0.003, 0.1 and 0.3 µg were not significantly different using a two-way ANOVA on the untransformed ELISA results (p=0.31). Whisker plot shows geometric mean and 95% confidence interval.


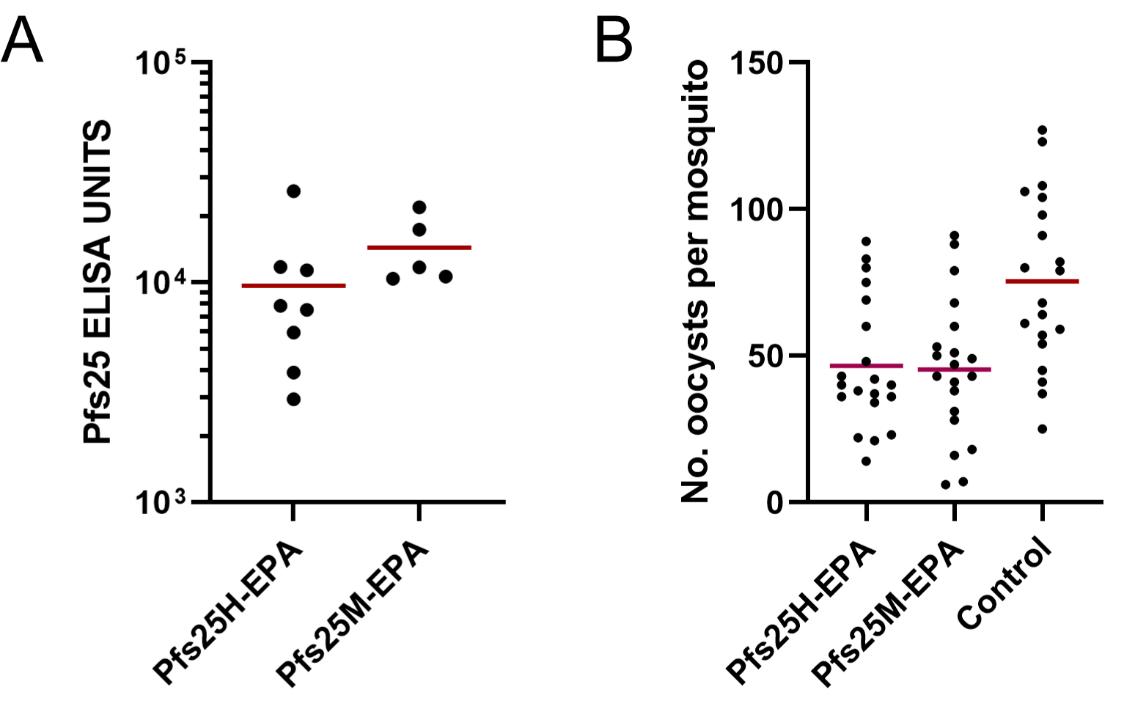


**Supplementary Figure 8. Assessment of Pfs25 ELISA titers of individual rhesus monkeys and TRA of pooled Pfs25H-EPA or Pfs25M-EPA specific rhesus IgG.** Rhesus antibody titers, reported as ELISA units, two weeks following a third immunization with Pfs25H-EPA/Alhydrogel or Pfs25M-EPA/Alhydrogel (A). Pooled purified IgG (0.93 mg/mL) was compared to pooled pre-immune IgG (0.93 mg/mL) in the standard membrane feeding assay (SMFA) (B). The average inhibitions of oocyst density for Pfs25H-EPA and Pfs25M-EPA were 38.4% and 39.9%, respectively using rhesus pre-immune IgG (0%) as a control (*p* < 0.001, Mann-Whitney test). The immunization schedules differed slightly. Pfs25H-EPA was administered on days 0, 56 and 112 while Pfs25M-EPA was administered on days 0, 56, 168. All assays included human complement. The Pfs25 geometric mean ELISA titers were not significantly different (*p* = 0.81) by Mann-Whitney test.


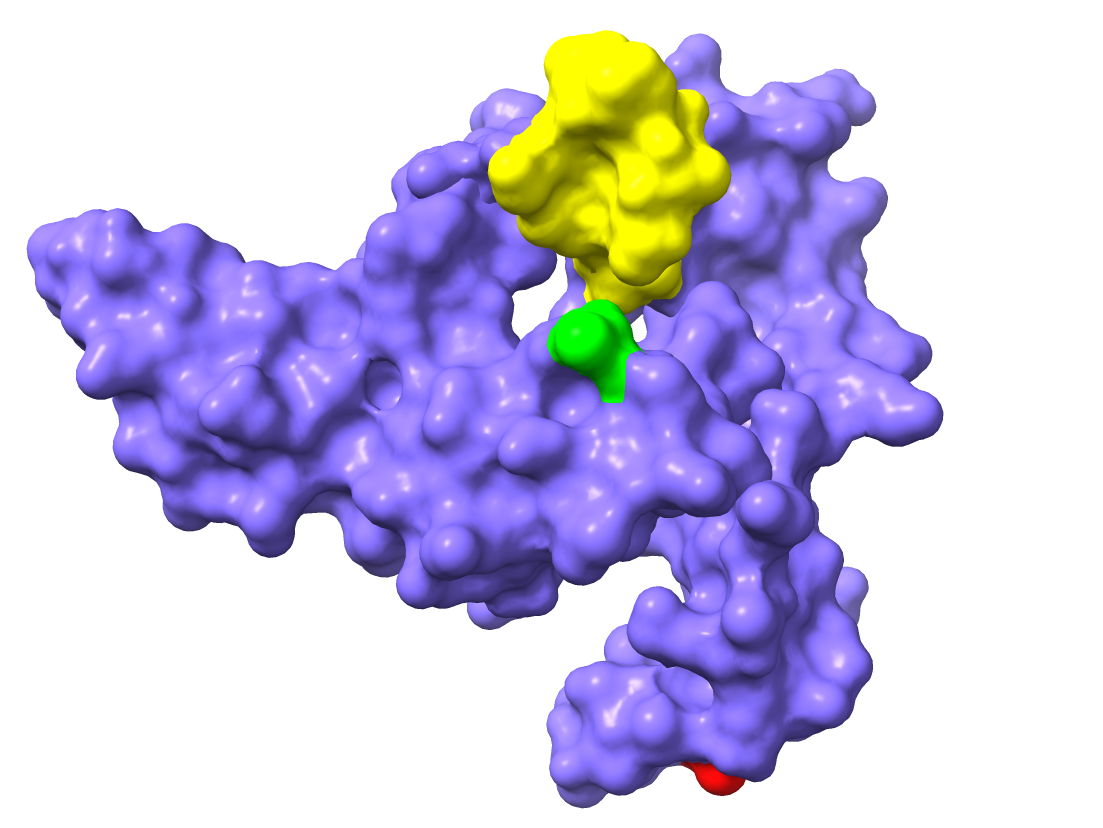


**Supplementary Figure 9. Surface model of Pfs25 structure showing the position of the amino- and carboxyl-terminal ends.** The amino-terminal K23 is colored green, and the carboxyl-terminal T193 is colored red. The 4B7 epitope is shown in yellow. The surface of PDB code 6PHF chain E was used.

**Supplementary Figure 10. Stereo view of a 2mFo–DFc electron density map contoured at 1.2 sigma.** The map includes electron density at the Pfs25M/Fab interface that is within 4 Å from Glu-60 of Pfs25M.
